# Effects of intra-aortic balloon pump on in-hospital outcomes and 1-year mortality in patients with acute myocardial infarction complicated by cardiogenic shock

**DOI:** 10.1186/s12872-023-03465-8

**Published:** 2023-08-29

**Authors:** Dingfeng Fang, Dongdong Yu, Jiabin Xu, Wei Ma, Yuxiang Zhong, Haibo Chen

**Affiliations:** 1 Shenzhen University Health Science Center, Shenzhen, 518060 China; 2 Department of Cardiology, Shenzhen Second People’s Hospital, No. 3002, Sungang West Road, Futian District, Shenzhen, 518035 China

**Keywords:** Intra-aortic balloon pump, Acute myocardial infarction, Cardiogenic shock, Mortality, Percutaneous coronary intervention

## Abstract

**Background:**

The role of intra-aortic balloon counterpulsation (IABP) in cardiogenic shock complicating acute myocardial infarction (AMI) is still a subject of intense debate. In this study, we aim to investigate the effect of IABP on the clinical outcomes of patients with AMI complicated by cardiogenic shock undergoing percutaneous coronary intervention (PCI).

**Methods:**

From the Medical Information Mart for Intensive Care (MIMIC)-IV 2.2, 6017 AMI patients were subtracted, and 250 patients with AMI complicated by cardiogenic shock undergoing PCI were analyzed. In-hospital outcomes (death, 24-hour urine volumes, length of ICU stays, and length of hospital stays) and 1-year mortality were compared between IABP and control during the hospital course and 12-month follow-up.

**Results:**

An IABP was implanted in 30.8% (77/250) of patients with infarct-related cardiogenic shock undergoing PCI. IABP patients had higher levels of Troponin T (3.94 [0.73–11.85] ng/ml vs. 1.99 [0.55–5.75] ng/ml, *p*-value = 0.02). IABP patients have a longer length of ICU and hospital stays (124 [63–212] hours vs. 83 [43–163] hours, *p*-value = 0.005; 250 [128–435] hours vs. 170 [86–294] hours, *p*-value = 0.009). IABP use was not associated with lower in-hospital mortality (33.8% vs. 33.0%, *p*-value = 0.90) and increased 24-hour urine volumes (2100 [1455–3208] ml vs. 1915 [1110–2815] ml, *p*-value = 0.25). In addition, 1-year mortality was not different between the IABP and the control group (48.1% vs. 48.0%; hazard ratio 1.04, 95% CI 0.70–1.54, *p*-value = 0.851).

**Conclusion:**

IABP may be associated with longer ICU and hospital stays but not better short-and long-term clinical prognosis.

**Supplementary Information:**

The online version contains supplementary material available at 10.1186/s12872-023-03465-8.

## Introduction

Cardiogenic shock is a life-threatening complication of acute myocardial infarction (AMI) in nearly 5-10% of patients [1]. The mortality of AMI complicated by cardiogenic shock remain unacceptably high at rates between 40 and 60% even when the patients undergo early revascularization [2–4]. Intra-aortic balloon pump (IABP) has been the most widely used percutaneous mechanical circulatory support (PMCS) device for several decades. The effects of IABP are believed to increase the myocardial oxygen supply/demand ratio and thus improve prognosis. Because registry studies indicated mortality benefits, former U.S. and European guidelines gave a class I.B. and class I.C. recommendation favoring IABP in patients with AMI complicated by cardiogenic shock [5–7]. However, the results of the largest randomized trial (the IABP-SHOCK-II [Intra-aortic Balloon Pump in Cardiogenic Shock-II study]) showed that IABP counterpulsation did not reduce 30­day, 1­year and 6-year mortality in cardiogenic shock complicating AMI undergoing early revascularization [8–10]. For this reason, the routine use of IABP in patients with infarct-related cardiogenic shock is no longer recommended by international guidelines [11, 12]. Unfortunately, the effective alternative PMCS devices for infarct-related cardiogenic shock are very limited. Therefore, the use of IABP was continued despite the paucity of survival benefit evidence based on randomized clinical trials [8–10, 13]. This study was designed to test the hypothesis that IABP can reduce mortality among patients with AMI complicated by cardiogenic shock undergoing percutaneous coronary intervention (PCI).

## Materials and methods

### Data source

This research was performed on a large critical-care database, namely, Medical Information Mart for Intensive Care (MIMIC)-IV, which comprised critical care data for patients admitted to intensive care units at the Beth Israel Deaconess Medical Center (BIDMC) [14, 15]. The latest version, MIMIC-IV 2.2, was updated in January 2023 and contained comprehensive clinical and laboratory data of patients. The date of death is determined by state and hospital records. If both exist, hospital records are used. MIMIC-IV collected state and hospital records for the date of death two years after the last patient discharge, which could lessen the impact of reporting delays in the date of death. The first author (DF) of this study passed the Protecting Human Research Participants exam (certification number: 50,924,352) to obtain the utility of the database. Data extraction from the database was done using the structured query language (SQL).

### Population selection criteria

**Fig. 1:**
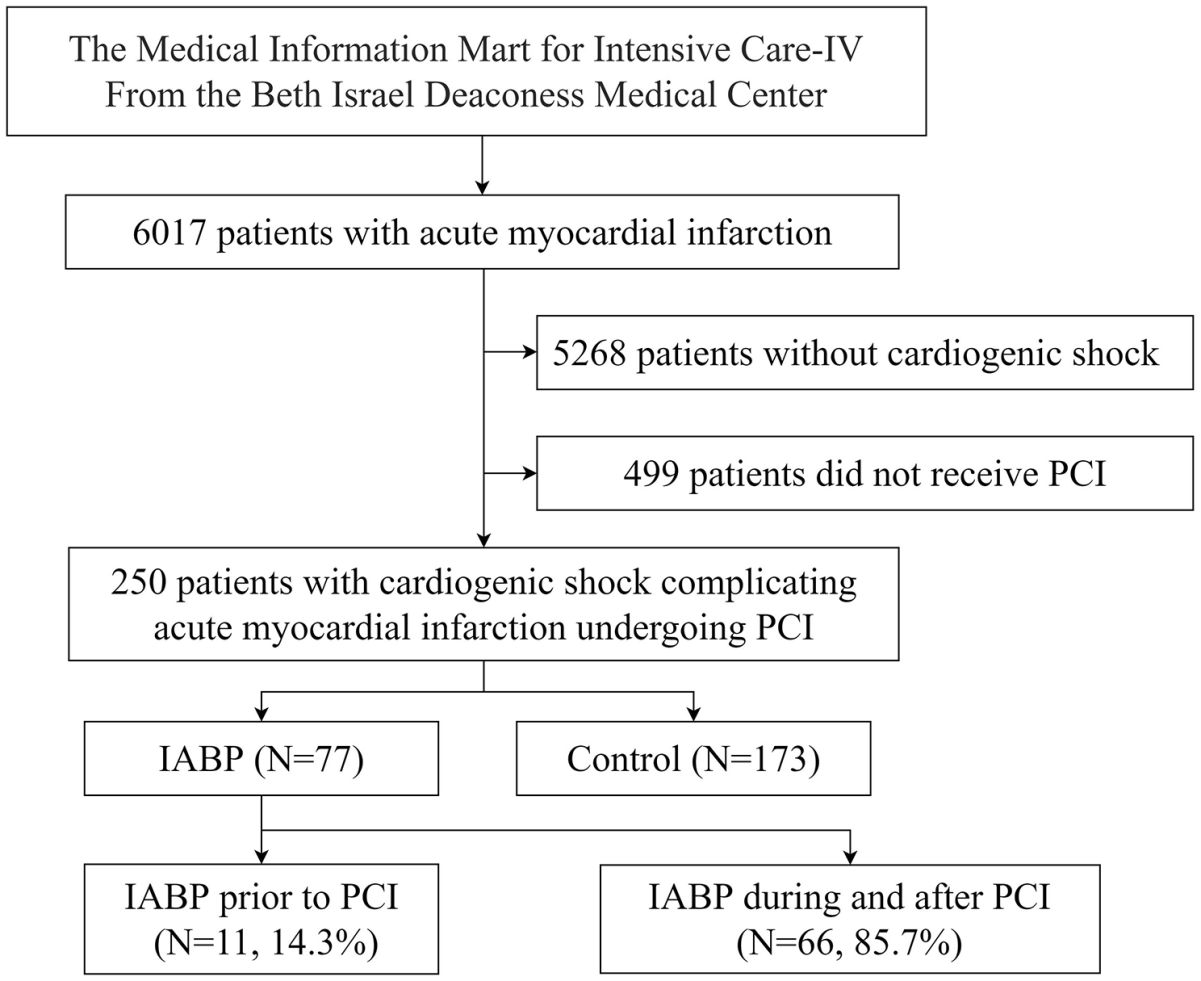
Flowchart of inclusion and exclusion of this study PCI = percutaneous coronary intervention, IABP = intra-aortic balloon pump

Patients with acute myocardial infarction admitted for the first time were included. Patients without infarct-related cardiogenic shock and those without percutaneous coronary intervention were excluded from the study. The flowchart of population selection is displayed in Fig. 1.

### Outcomes and covariates

The extraction variables included age, gender, diagnosis of STEMI, diagnosis of chronic total occlusion (CTO), history (hypertension, diabetes, tobacco, prior myocardial infarction, prior chronic kidney disease), arterial blood gas on arrival (pH, partial pressure of oxygen [PaO_2_], partial pressure of carbon dioxide [PaCO_2_], lactate), baseline serum creatinine, hemoglobin, total cholesterol (T.C.), high-density lipoprotein cholesterol (HDL-C), low-density lipoprotein cholesterol (LDL-C), troponin T, and mechanical ventilation. The primary outcome was one-year (long-term) mortality. The secondary outcomes included in-hospital (short-term) mortality, 24-hour urine volumes, length of intensive care unit (ICU) stay, and length of hospital stay. Patients with cardiogenic shock complicating AMI undergoing PCI were distributed to IABP versus control. The start and end times of IABP were also extracted to calculate the IABP duration and compare it with the PCI procedure time.

### Statistical analyses

Skewness and kurtosis tests were used to test the normality of continuous variables. Normally distributed continuous variables were compared using Student *t-*tests and were expressed as mean ± S.D. Skewed distributed continuous variables were compared using a two-sample Wilcoxon rank-sum (Mann-Whitney) test and were expressed as median (interquartile range). Categorical variables were compared using Pearson chi-square tests and were expressed as numbers (percentages). Survival probability throughout one year after admission was characterized using Kaplan–Meier survival estimates, with the log-rank test used to compare the two groups.

Identifying independent clinical and laboratory risk factors at baseline related to death was performed using Cox proportional hazards regression modeling. All variables considered clinically relevant and related to mortality on univariable analysis (defined by P < 0.10) were further analyzed in a stepwise multivariable model.

STATA (version 17.0, USA) software was used for the statistical analysis. All calculated *p*-value were 2-sided, and *p*-value < 0.05 were considered statistically significant.

## Results

In our study, 6017 patients with AMI were extracted, and 250 patients with AMI complicated by cardiogenic shock undergoing PCI were included, and the associated flow chart is displayed in Fig. 1. The cardiogenic shock occurred in 12.4% (749/6017) of patients with AMI. An IABP was implanted in 30.8% (77/250) of patients. The baseline characteristics of patients treated with and without IABP are presented in Table 1. Patients managed with IABP had a higher level of Troponin T (3.94 [0.73–11.85] ng/ml vs. 1.99 [0.55–5.75] ng/ml, *p*-value = 0.02). Other baseline characteristics were similar between the groups. IABP placement was performed after PCI in 85.7% of patients. The mean IABP duration was 69.6 h in all patients managed with IABP.

**Fig. 2:**
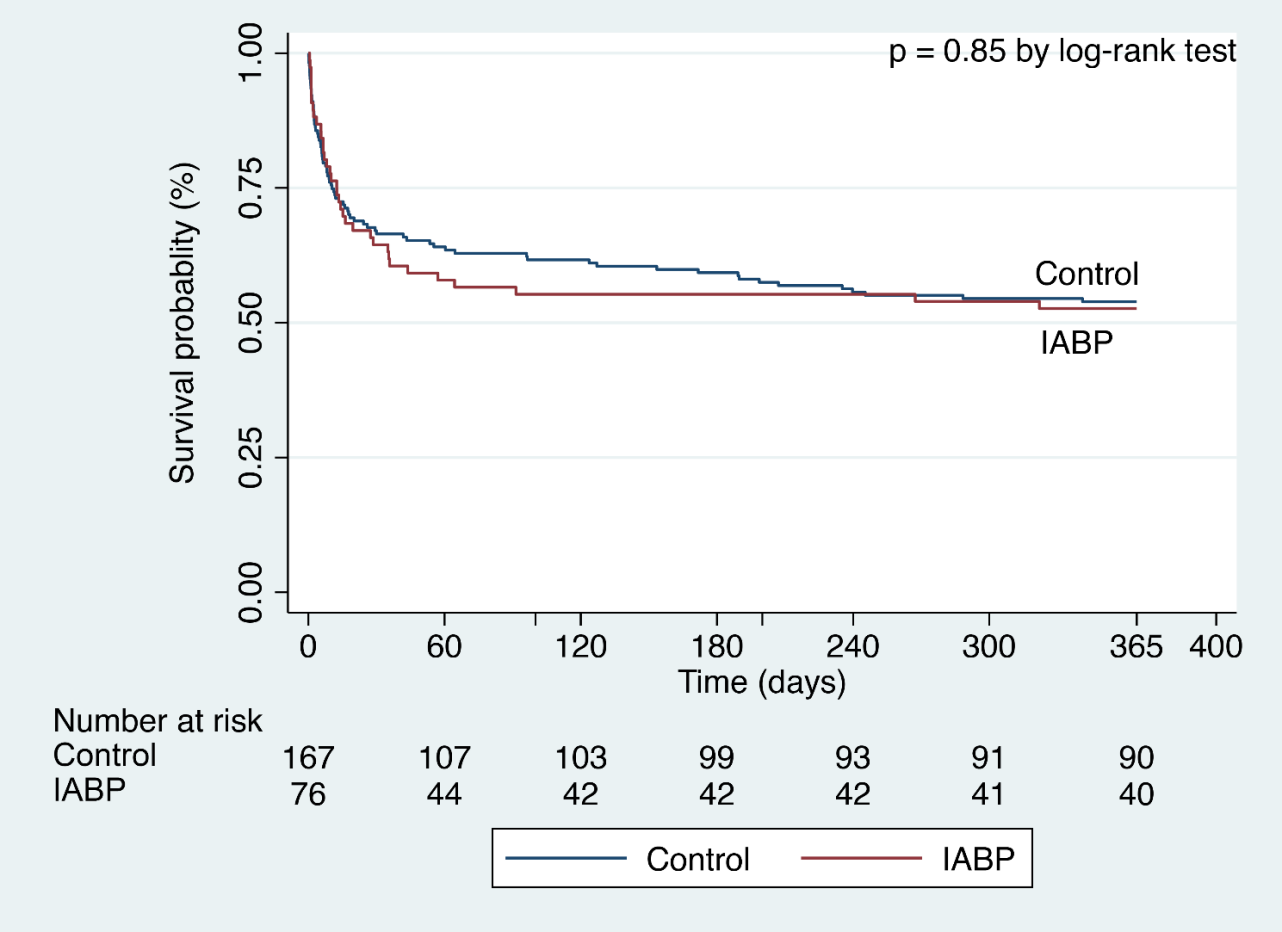
Time-to-survival curves through 1 year Time-to-survival curves through 1 year for all-cause mortality. The cumulative 1-year (long-term) survival of patients managed with and without IABP was characterized using Kaplan-Meier estimates. The *p*-value was calculated by the log-rank test. IABP indicates intra-aortic balloon pump

The clinical outcomes of patients treated with and without IABP are displayed in Table 2. In-hospital (short-term) and 1-year (long-term) mortality was similar in the patients between the two groups (33.8% vs. 33.0%, *p*-value = 0.90; 48.1% vs. 48.0%, *p*-value = 0.99). 24-hour urine volumes on the first day were similar in the patients receiving an IABP and those who did not (2100 [1455–3208] ml vs. 1915 [1110–2815] ml, *p*-value = 0.25). Patients managed with IABP were more likely to have a longer length of ICU and hospital stays (124 [63–212] hours vs. 83 [43–163] hours, *p*-value = 0.005; 250 [128–435] hours vs. 170 [86–294] hours, *p*-value = 0.009). The cumulative 1-year (long-term) survival of patients managed with and without IABP was similar (*p*-value = 0.85). Time-to-survival curves through 1 year are shown in Fig. 2.

Stepwise multivariable modeling revealed increasing age, mechanical ventilation, chronic total occlusion, and baseline arterial lactate as independent risk factors for long-term mortality (Table 2). IABP treatment was not predictive of survival (HR 1.04, 95% CI 0.70–1.54, *p*-value = 0.851). The timing of IABP implantation and duration of IABP treatment was also not predictive of long-term outcomes.

**Table 1:** Baseline characteristics of patients treated with and without IABP

	IABP (n = 77)	Control (n = 173)	*p*-value
Age (year)	70 (64–77)	70 (61–79)	0.80
Female gender	30 (39.0%)	60 (36.4%)	0.70
STEMI	64 (83.1%)	129 (74.6%)	0.14
NSTEMI	13 (16.9%)	44 (25.4%)	0.14
CTO	13 (16.9%)	19 (11.0%)	0.20
Hypertension	29 (37.7%)	48 (27.7%)	0.12
Diabetes	34 (44.1%)	58 (33.5%)	0.11
Smoker	9 (11.7%)	10 (5.7%)	0.10
Prior MI	12 (15.6%)	22 (12.7%)	0.54
Prior CKD	22 (28.6%)	49 (28.3%)	0.97
Arterial blood gas			
pH	7.31 (7.23–7.39)	7.32 (7.25–7.40)	0.65
PaO2 (mmHg)	104 (63–168)	96 (61–193)	0.82
PaCO2 (mmHg)	40 (34–48)	41 (35–48)	0.25
Lactate (mmol/liter)	2.4 (1.5–3.5)	2.1 (1.5–3.8)	0.98
Serum creatinine (mg/dl)	0.8 (0.7–1.1)	0.9 (0.7–1.1)	0.24
Hemoglobin (g/dl)	11.9 ± 2.4	11.7 ± 2.3	0.64
TC (mg/dl)	138.5 (110.0-164.0)	146.5 (117.0-176.5)	0.59
HDL-C (mg/dl)	37.6 ± 16.8	39.6 ± 13.0	0.62
LDL-C (mg/dl)	96.5 ± 36.0	91.0 ± 36.3	0.70
Troponin T (ng/ml)	3.94 (0.73–11.85)	1.99 (0.55–5.75)	0.02
Mechanical ventilation	49 (63.6%)	89 (51.4%)	0.07

STEMI = S.T. segment elevation myocardial infarction, NSTEMI = non-ST segment elevation myocardial infarction, CTO = chronic total occlusion, MI = myocardial infarction, CKD = chronic kidney disease, PaO_2_ = partial pressure of oxygen, PaCO_2_ = partial pressure of carbon dioxide, TC = total cholesterol, HDL-C = high-density lipoprotein cholesterol, LDL-C = low-density lipoprotein cholesterol

**Table 2:** Clinical outcomes of patients treated with and without IABP

	IABP (n = 77)	Control (n = 173)	*p*-value
In-hospital death	26 (33.8%)	57 (33.0%)	0.90
Death during 1 year	37 (48.1%)	83 (48.0%)	0.99
24-hour urine volumes (ml)	2100 (1455–3208)	1915 (1110–2815)	0.25
Length of ICU stay (hour)	124 (63–212)	83 (43–163)	0.005
Length of hospital stay (hour)	250 (128–435)	170 (86–294)	0.009

ICU = intensive care unit

Time-to-survival curves through 1 year for all-cause mortality. The cumulative 1-year (long-term) survival of patients managed with and without IABP was characterized using Kaplan-Meier estimates. The *p*-value was calculated by the log-rank test. IABP indicates intra-aortic balloon pump.

**Table 3:** Predictors of 1-year mortality in univariable and stepwise multivariable Cox regression analysis

Variable	Univariable		Stepwise Multivariable	
	Hazard Ratio (95% CI)	*p*-Value	Hazard Ratio (95% CI)	*p*-Value
IABP	1.04 (0.70–1.54)	0.851	-	-
IABP before PCI	1.19 (0.50–2.87)	0.693	-	-
Duration of IABP	1.00 (0.99–1.01)	0.891	-	-
STEMI	1.21 (0.78–1.89)	0.399	-	-
Hypertension	1.04 (0.70–1.54)	0.841	-	-
Diabetes	1.14 (0.78–1.67)	0.484	-	-
Smoker	0.70 (0.33–1.5)	0.358	-	-
Prior MI	0.93 (0.53–1.63)	0.799	-	-
Prior CKD	1.77 (1.21–2.59)	0.003	-	-
pH, < 7.30 at admission	1.47 (1.02–2.15)	0.041	-	-
Baseline arterial PaO2, mmHg	1.00 (0.99-1.00)	0.930	-	-
Baseline atrial PaCO2, mmHg	1.01 (0.99–1.03)	0.528	-	-
Hemoglobin, mg/dl	0.87 (0.80–0.95)	0.001	-	-
LDL-C, mg/ml	0.99 (0.97–1.01)	0.475	-	-
Troponin T, ng/ml	1.02 (0.99–1.04)	0.115	-	-
Baseline serum creatinine, mg/dl	1.33 (1.12–1.57)	0.001	-	-
Age, year	1.03 (1.01–1.05)	< 0.001	1.022 (1.00-1.04)	0.022
Mechanical ventilation	2.27 (1.53–3.37)	< 0.001	2.24 (1.39–3.61)	0.001
CTO	1.69 (1.03–2.76)	0.038	2.39 (1.36–4.19)	0.002
Baseline lactate, mmol/liter	1.22 (1.15–1.30)	< 0.001	1.23 (1.15–1.31)	< 0.001

Baseline variables related to 1-year mortality on univariable analysis are defined by a *p*-value < 0.10. The first 16 variables initially entered into the model were not independently associated with mortality in the stepwise multivariable model. IABP = intra-aortic balloon pump, PCI = percutaneous coronary intervention, STEMI = ST-segment elevation myocardial infarction, MI = myocardial infarction, CKD = chronic kidney disease, PaO2 = partial pressure of oxygen, PaCO2 = partial pressure of carbon dioxide, LDL-C = low-density lipoprotein cholesterol, CTO = chronic total occlusion

## Discussion

In this study, patients with AMI complicated by cardiogenic shock remain at a high risk of mortality (approximately 50%) regardless of whether they receive IABP treatment. Early studies indicate a mortality benefit for several indications, including AMI complicated by cardiogenic shock, to support coronary artery bypass graft (CABG) or high-risk PCI, as well as following thrombolysis [16–18]. However, much debate has been on whether IABP counterpulsation benefits patients with cardiogenic shock. Indeed, the multicenter open-label IABP-SHOCK II trial’s only adequately powered randomized trial failed to show any mortality benefit for IABP over the control or any advantages for secondary outcomes [8–10]. Based on IABP-SHOCK, current guidelines recommend not using IABP routinely [11, 12]. In the present study, IABP was not associated with survival benefits, which is consistent with the results of the IABP-SHOCK-II trial. However, in this study, patients managed with IABP were more likely to have higher troponin T levels, indicating that these patients were admitted later and had larger infarct sizes. In addition, in the present study, IABP use was seemly greater in patients with higher risk characteristics, including those with STEMI (83.1% vs. 74.6%), CTO (16.9% vs. 11.0%), a history of hypertension (37.7% vs. 27.7%), a history of diabetes (44.1% vs. 33.5%), a history of tobacco (11.7% vs. 5.7%), prior MI (15.6% vs. 12.7%), and higher arterial lactate (2.4 mmol/liter vs. 2.1 mmol/liter) and in patients supported by mechanical ventilation (63.6% vs. 51.4%) but those characteristics without significantly statistical differences (p-value < 0.05; Table 1). In stepwise multivariable Cox regression analysis, age, mechanical ventilation, CTO, and baseline lactate were associated with higher long-term mortality (Table 3). Moreover, the use of IABP was associated with longer ICU and hospital stays (124 [63–212] vs. 83 [43–163], *p*-value = 0.005; 250 [128–435] vs. 170 [86–294], *p*-value = 0.009). Therefore, in this study, the baseline characteristics were not remarkably dissimilar between patients with IABP and controls. The short-term and long-term mortality was similar between patients managed with IABP and control, which may not be enough evidence to suggest that patients with infarct-related cardiogenic shock can benefit from IABP treatment.

In the present study, approximately one-third of patients with AMI complicated by cardiogenic shock managed with IABP. Indeed, however, the application of IABP in cardiogenic shock was significantly influenced by the results of IABP-SHOCK. Based on IABP-SHOCK, the use of IABP has decreased dramatically since 2012, despite increasing rates of infarct-related cardiogenic shock [19]. Despite several pathophysiological studies suggesting a hemodynamic gain under IABP for cardiogenic shock [20, 21], only one randomized trial, IABP-SHOCK, indicates no improvement was observed in measured hemodynamic parameters [22]. Under some clinical situations, the ventricular-arterial uncoupling may cause the IABP’s inefficiency and affect IABP performance via complex mechanisms [23]. IABP was widely used clinically, mainly based on its underlying functional idea to increase left ventricular output and coronary perfusion by diastolic augmentation and afterload reduction. It is probably unreasonable that there were no differences in median diastolic pressure between patients managed with IABP and controls in the IABP-SHOCK II trial (55 [46–67] mmHg vs. 55 [45–65]mmHg) [8]. In addition, it is important to remember that patients without adequate myocardial reperfusion may benefit from the increased coronary perfusion pressure obtained by IABP, whereas this effect of IABP would not translate into a therapeutic advantage in patients with enough flow [23]. Hence, denying the benefits of IABP based on just one powered randomized trial is likely unreasonable. At the very least, no study found that IABP increased significant adverse events and mortality. Indeed, Impella Support, more advanced and expensive PMCS devices, was not associated with a mortality benefit compared with IABP [24–26]. In addition, Impella, compared with IABP, was associated with a higher rate of major bleeding events in randomized controlled trial [24]. In this regard, the continued use of IABP in patients with AMI complicated by cardiogenic shock is not unreasonable.

IABP seems to have other benefits besides increasing coronary perfusion and left ventricular output. A cohort study found that IABP is probably associated with improving cerebral blood flow, particularly in patients with impaired left cardiac function [27]. This study implicated that IABP is probably associated with improving neurological outcomes. In a clinical trial [28], IABP was found to have synergistic effects with extracorporeal membrane oxygenation (ECMO). Furthermore, it was found that IABP, in conjunction with ECMO, can increase in-hospital survival in patients with cardiogenic shock more efficiently than ECMO alone [29]. Studies have also found that IABP can increase renal blood flow [30]. However, the use of IABP in this study was not associated with a significant increase in 24-hour urine volumes compared to controls, suggesting that IABP is unlikely to improve renal perfusion significantly. What is certain, however, is that IABP does not cause kidney injury.

Managing infarct-related cardiogenic shock is challenging, despite the rapid development of primary PCI. Cardiologists face a significant conundrum because there are no evidence-based alternative interventions for patients with AMI complicated by cardiogenic shock, who have rapidly deteriorating hemodynamics and bad clinical outcomes. As a result, we urgently require a more advanced strategy to improve the poor clinical outcomes of infarct-related cardiogenic shock patients. Before it, IABP seemed to be the only intervention available for patients at most institutions; it seemly could explain why the IABP was still used in many hospitals despite the current guidelines recommend not to use it routinely. However, it is well known that IABP is associated with more complications (major limb ischemia, severe bleeding, balloon leak, death directly due to IABP insertion or failure) and higher medical costs [31]. Whether to use IABP in patients with myocardial infarction complicated by cardiogenic shock is probably based on the operator’s clinical experience in most hospitals. From this study, we prefer the current guideline recommendation not to use IABP routinely [11, 12].

Our study’s primary limitation is that it is a retrospective observational study without randomization. The results of this study are insufficient to conclude that IABP will benefit patients with AMI complicated by cardiogenic shock. However, in our study, only 14.3% of patients received treatment of IABP before the percutaneous coronary intervention, so the timing of the use of IABP probably impacted the findings. If the IABP was done before PCI, possible outcomes could be better. In addition, due to the lack of some data, indicators such as hemodynamic parameters focused on by some researchers were not included.

## Conclusion

In summary, even when patients with AMI complicated by cardiogenic shock receive percutaneous coronary intervention, the 1-year mortality rate remains unacceptably high (about 50%). The results of this study are consistent with IABP-SHOCK in that IABP is not a predictor of both short- and long-term survival. IABP patients tended to have longer length of ICU and hospital stays. To improve the clinical outcomes of patients with AMI complicated by cardiogenic shock, more advanced evidence-based PMCS devices are urgently required.

### Electronic supplementary material

Below is the link to the electronic supplementary material.

Additional File 1: CITI program

## Data Availability

All data generated or analyzed during this study are included in this published article. After completing relevant training and registration, the raw data are available in the physionet (https://physionet.org/content/mimiciv) [14, 15].
