# Supplementary material for: Effects of intra-aortic balloon pump on in-hospital outcomes and 1-year mortality in patients with acute myocardial infarction complicated by cardiogenic shock
Source: BMC Cardiovasc Disord. 2023 Aug 29;23:425. doi: 10.1186/s12872-023-03465-8 (PMC10466728; doi:10.1186/s12872-023-03465-8)
Supplement: Supplementary file 1 — Additional File 1: CITI program [file 12872_2023_3465_MOESM1_ESM.pdf]

CITI PROGRAM

Completion Date	27-Aug-2022
Expiration Date	26-Aug-2025
Record ID	50924352

This is to certify that:

Dingfeng Fang

Has completed the following CITI Program course:

Not valid for renewal of certification through CME.

Human Research
(Curriculum Group)
Data or Specimens Only Research
(Course Learner Group)
2 - Refresher Course
(Stage)

Under requirements set by:

Massachusetts Institute of Technology Affiliates

CITI
Collaborative Institutional Training Initiative

Verify at www.citiprogram.org/verify/?wf43c7d18-4428-451f-b981-443cb7ee74b8-50924352
